# Crystal structure of [NiHg(SCN)_4_(CH_3_OH)_2_]_*n*_

**DOI:** 10.1107/S1600536814009532

**Published:** 2014-07-19

**Authors:** Matthias Weil, Thomas Häusler

**Affiliations:** a Institute for Chemical Technologies and Analytics, Division of Structural Chemistry, Vienna University of Technology, Getreidemarkt 9/164-SC, A-1060 Vienna, Austria

**Keywords:** crystal structure, NLO materials, gel growth, nickel, mercury

## Abstract

The title compound, *catena*-poly[[bis­(methanol-κ*O*)nickel(II)]-di-μ-thio­cyanato-κ^4^ *N*:*S*-mercurate(II)-di-μ-thio­cyanato-κ^4^ *N*:*S*], was obtained from a gel-growth method using tetra­meth­oxy­silane as gelling agent. The crystal structure is composed of rather regular HgS_4_ tetra­hedra (point group symmetry .2.) and *trans*-NiN_4_O_2_ octa­hedra (point group symmetry 2..) that are linked through thio­cyanato bridges into a three-dimensional framework. The methanol mol­ecules coordinate *via* the O atom to the Ni^2+^ cations and point into the voids of this arrangement while a weak O—H⋯S hydrogen bond to an adjacent S atom stabilizes it.

## Abstract 2

The crystal structure of [NiHg(SCN)_4_(CH_3_OH)_2_]_*n*_ is made up of HgS_4_ tetra­hedra and *trans*-NiN_4_O_2_ octa­hedra, linked together by thio­cyanato bridges. The methanol mol­ecules point to the cavities of the resulting framework.

## Chemical context   

Compounds of the type *M*Hg(SCN)_4_ (*M* is a divalent trans­ition metal) exhibit inter­esting physical properties. For example, CoHg(SCN)_4_ is a calibrant for magnetic susceptibility measurements using the Faraday method (Brown *et al.*, 1977 ▶), and representatives with *M* = Fe, Mn, Zn and Cd show second-order non-linear optical (NLO) properties (Bergman *et al.*, 1970 ▶; Yan *et al.*, 1999 ▶).
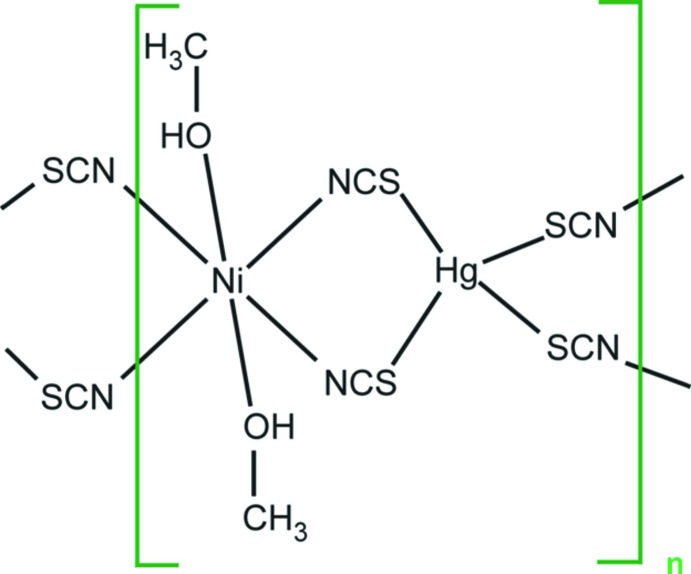


Most of the *M*Hg(SCN)_4_ compounds have been structurally characterized, including MnHg(SCN)_4_, FeHg(SCN)_4_ (Yan *et al.*, 1999 ▶), CoHg(SCN)_4_ (Jeffery & Rose, 1968 ▶), CuHg(SCN)_4_ (Porai Koshits, 1963 ▶; Khandar *et al.*, 2011 ▶), ZnHg(SCN)_4_ (Xu *et al.*, 1999 ▶) and CdHg(SCN)_4_ (Iizuka & Sudo, 1968 ▶). The crystal structure of NiHg(SCN)_4_ has not been reported up to now, and only the structures of the related hydrous phase [NiHg(SCN)_4_(H_2_O)_2_]_*n*_ (Porai Koshits, 1960 ▶) and of the mercury-richer phase NiHg_2_(SCN)_6_ (Iizuka, 1978 ▶) have been determined.

In an attempt to grow crystals of the desired compound NiHg(SCN)_4_ using a gel-growth method (Henisch, 1996 ▶), starting from TMOS (tetra­meth­oxy­silane) as gelling agent, we obtained the title compound, [NiHg(SCN)_4_(CH_3_OH)_2_]*_n_ viz.* a methanol-containing phase, instead. Methanol is generated during the gelling process of the silicate-based material according to the idealized reaction (H_3_CO)_4_Si + 4 H_2_O → 4 H_4_SiO_4_ + 4 H_3_COH and then becomes part of the crystal structure.

## Structural commentary   

**Figure 1:**
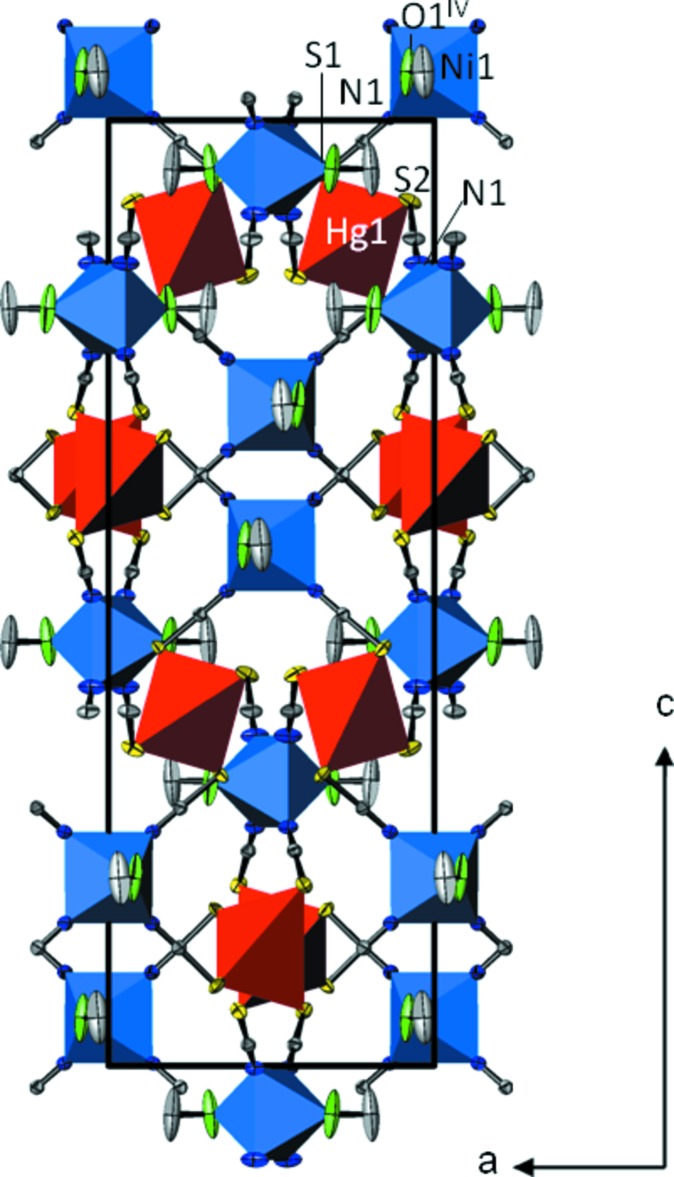
The crystal structure of [NiHg(SCN)_4_(CH_3_OH)_2_] in a projection along [010]. Displacement ellipsoids are drawn at the 90% probability level. H atoms are omitted for clarity. [Symmetry code: (iv) −*x*, −*y* + 1, *z*.]

The basic structure units of [NiHg(SCN)_4_(CH_3_OH)_2_]_*n*_ are HgS_4_ tetra­hedra (point group symmetry .2.) and *trans*-NiN_4_O_2_ octa­hedra (point group symmetry 2..) that are linked through the bridging thio­cyanate anions into a three-dimensional framework structure (Fig. 1 ▶). The Hg—S bond lengths [mean 2.552 (3) Å; Table 1 ▶] are in very good agreement compared with those of HgS_4_ tetra­hedra in the above-mentioned solvent-free *M*Hg(SCN)_4_ structures, which have a mean of 2.57 (5) Å. The *trans*-NiN_4_O_2_ octa­hedra are defined by four N atoms belonging to four bridging thio­cyanate anions and by two O atoms of isolated methanol mol­ecules. The displacement parameters of the methanol mol­ecule are rather high. The methanol mol­ecule has relatively much space for libration, because it is not part of the framework structure and points into the remaining free space. Thus the displacement ellipsoids of the methanol O and especially of the C atom are enlarged (Fig. 1 ▶). Moreover, there is only a weak hydrogen-bonding inter­action to an adjacent S atom that stabilizes this arrangement (Table 2 ▶).

**Figure 2:**
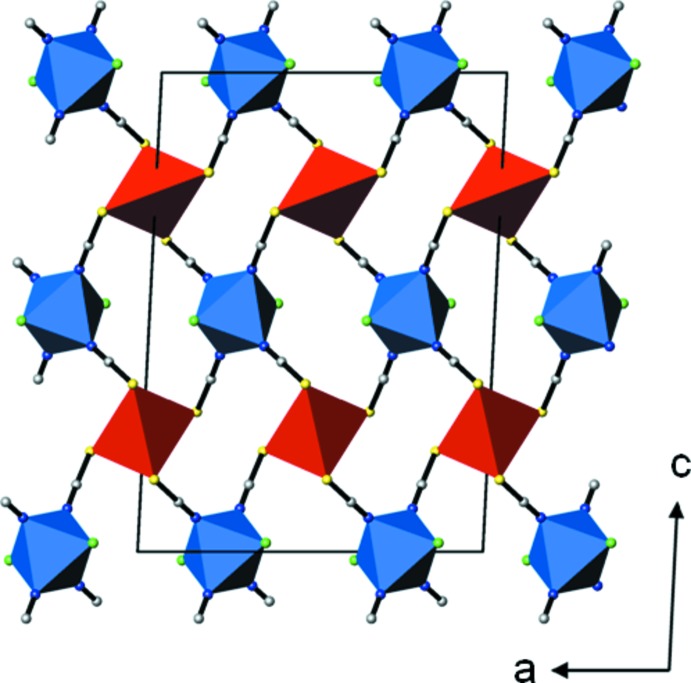
The crystal structure of [NiHg(SCN)_4_(H_2_O)_2_] (Porai Koshits, 1960 ▶) in a projection along [010]. Colour code as in Fig. 1 ▶.

[NiHg(SCN)_4_(CH_3_OH)_2_]_*n*_ and [NiHg(SCN)_4_(H_2_O)_2_]_*n*_ have a similar composition. Although the basic structure units (HgS_4_ tetra­hedra and *trans*-NiN_4_O_2_ octa­hedra linked by thio­cyanate bridges) are the same, the corresponding crystal structures are markedly different. The methanol-containing structure has tetra­gonal symmetry and is non-centrosymmetric, the water-containing structure has monoclinic symmetry and is centrosymmetric (space group *C*2/*c*). Whereas in the water-containing structure the HgS_4_ and NiN_4_O_2_ polyhedra are alternately arranged in layers parallel to (001) (Fig. 2 ▶), the arrangement in the methanol-containing compound is markedly different (Fig. 1 ▶).

The common structural motif in the above-mentioned *M*Hg(SCN)_4_ compounds is the linkage of *M*N_4_ units (planar configuration for Cu and tetrahedral for all other *M* members) and tetra­hedral HgS_4_ units through thio­cyanate bridges. It seems that a coordination number of four is not favoured for structures with *M* = Ni. In the structures of [NiHg(SCN)_4_(CH_3_OH)_2_]_*n*_, [NiHg(SCN)_4_(H_2_O)_2_]_*n*_ and NiHg_2_(SCN)_6_, the Ni^2+^ ions all have coordination numbers of six, which is probably the reason why a compound with composition NiHg(SCN)_4_ (most probably requiring a [4]-coordination for Ni^2+^) has not yet been isolated.

## Synthesis and crystallization   

Hg(SCN)_2_ was prepared by adding stoichiometric amounts of KSCN to a slightly acidified aqueous solution of Hg(NO_3_)_2_. The colourless precipitate was filtered off, washed with water and dried.

For the gel-growth experiment, 1.2 g Ni(NO_3_)_2_·6H_2_O and 1.2 g NH_4_SCN were dissolved in 20 ml water. To this solution, 0.5 g freshly prepared Hg(SCN)_2_ was slowly added until complete dissolution. Then 2 ml TMOS was added dropwise under stirring. Gelling time was about 3 h. After one week, blue single crystals of the title compound up to 5 mm in length had formed in the gel matrix.

## Refinement   

The H atom of the methanol hy­droxy group was located from a difference map and was refined with a distance restraint of 0.90 (1) Å. The H atoms associated with the methyl group of the methanol mol­ecule could not be located from difference Fourier maps. As a result of the high libration of this mol­ecule, it seems probable that the methyl H atoms are disordered and were therefore refined with two positions with half-occupancy and rotated by 60 degrees. *U*_eq_ of these H atoms were set 1.5*U*_iso_ of the parent C atom. The remaining maximum and minimum electron densities are found 0.36 and 0.06 Å, respectively, from atom O1. Reflection (011) was affected by the beamstop and was discarded from the refinement. Experimental details are given in Table 3 ▶.

## Supplementary Material

Crystal structure: contains datablock(s) I, global. DOI: 10.1107/S1600536814009532/hb0003sup1.cif

Structure factors: contains datablock(s) I. DOI: 10.1107/S1600536814009532/hb0003Isup2.hkl

CCDC reference: 1004260

Additional supporting information:  crystallographic information; 3D view; checkCIF report

## Figures and Tables

**Table 1:** Selected bond lengths (Å)

Hg1—S1^i^	2.5499 (7)	Ni1—N2^iii^	2.041 (2)
Hg1—S1	2.5499 (7)	Ni1—N1^iv^	2.045 (2)
Hg1—S2	2.5546 (7)	Ni1—N1	2.045 (2)
Hg1—S2^i^	2.5546 (7)	Ni1—O1	2.066 (2)
Ni1—N2^ii^	2.041 (2)	Ni1—O1^iv^	2.066 (2)

Symmetry codes: (i) 
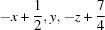
; (ii) 
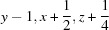
; (iii) 
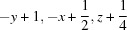
; (iv) 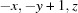.

**Table 2:** Hydrogen-bond geometry (Å, °)

*D*—H⋯*A*	*D*—H	H⋯*A*	*D*⋯*A*	*D*—H⋯*A*
O1—H1⋯S1^v^	0.90 (1)	2.40 (2)	3.262 (2)	160 (4)

Symmetry code: (v) 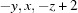.

**Table 3:** Experimental details

Crystal data	
Chemical formula	[NiHg(NCS)_4_(CH_4_O)_2_]
*M*_r_	555.70
Crystal system, space group	Tetragonal, *I* 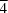 2*d*
Temperature (K)	100
*a*, *c* (Å)	10.1746 (3), 29.5107 (11)
*V* (Å^3^)	3055.02 (17)
*Z*	8
Radiation type	Mo *K*α
μ (mm^−1^)	11.81
Crystal size (mm)	0.18 × 0.18 × 0.18

Data collection	
Diffractometer	Bruker APEXII CCD
Absorption correction	Multi-scan (*SADABS*; Bruker, 2008 ▶)
*T*_min_, *T*_max_	0.567, 0.748
No. of measured, independent and observed [*I* > 2σ(*I*)] reflections	17699, 4684, 4214
*R*_int_	0.028
(sin θ/λ)_max_ (Å^−1^)	0.904

Refinement	
*R*[*F* ^2^ > 2σ(*F* ^2^)], *wR*(*F* ^2^), *S*	0.026, 0.051, 0.96
No. of reflections	4684
No. of parameters	87
H-atom treatment	H atoms treated by a mixture of independent and constrained refinement
Δρ_max_, Δρ_min_ (e Å^−3^)	1.63, −1.46
Absolute structure	Flack (1983 ▶), 2098 Friedel pairs
Absolute structure parameter	0.011 (4)

Computer programs: *APEX2* and *SAINT* (Bruker, 2008 ▶), *SHELXS97* and *SHELXL97* (Sheldrick, 2008 ▶) and *ATOMS for Windows* (Dowty, 2006 ▶).

## supplementary crystallographic information

### Crystal data

**Table:**

[NiHg(NCS)_4_(CH_4_O)_2_]	*D*_x_ = 2.416 Mg m^−^^3^
*M**_r_* = 555.70	Mo *K*α radiation, λ = 0.71073 Å
Tetragonal, *I*42*d*	Cell parameters from 9082 reflections
Hall symbol: I -4 2bw	θ = 2.9–40.0°
*a* = 10.1746 (3) Å	µ = 11.81 mm^−^^1^
*c* = 29.5107 (11) Å	*T* = 100 K
*V* = 3055.02 (17) Å^3^	Spherical, blue
*Z* = 8	0.18 × 0.18 × 0.18 mm
*F*(000) = 2080	

### Data collection

**Table:**

Bruker APEXII CCD diffractometer	4684 independent reflections
Radiation source: fine-focus sealed tube	4214 reflections with *I* > 2σ(*I*)
Graphite monochromator	*R*_int_ = 0.028
ω and φ scans	θ_max_ = 40.0°, θ_min_ = 2.8°
Absorption correction: multi-scan (*SADABS*; Bruker, 2008)	*h* = −17→17
*T*_min_ = 0.567, *T*_max_ = 0.748	*k* = −18→12
17699 measured reflections	*l* = −41→53

### Refinement

**Table:**

Refinement on *F*^2^	Secondary atom site location: difference Fourier map
Least-squares matrix: full	Hydrogen site location: inferred from neighbouring sites
*R*[*F*^2^ > 2σ(*F*^2^)] = 0.026	H atoms treated by a mixture of independent and constrained refinement
*wR*(*F*^2^) = 0.051	*w* = 1/[σ^2^(*F*_o_^2^) + (0.*P*)^2^] where *P* = (*F*_o_^2^ + 2*F*_c_^2^)/3
*S* = 0.96	(Δ/σ)_max_ = 0.004
4684 reflections	Δρ_max_ = 1.63 e Å^−^^3^
87 parameters	Δρ_min_ = −1.46 e Å^−^^3^
0 restraints	Absolute structure: Flack (1983), 2098 Friedel pairs
Primary atom site location: structure-invariant direct methods	Absolute structure parameter: 0.011 (4)

### Special details

**Table:**

Geometry. All e.s.d.'s (except the e.s.d. in the dihedral angle between two l.s. planes) are estimated using the full covariance matrix. The cell e.s.d.'s are taken into account individually in the estimation of e.s.d.'s in distances, angles and torsion angles; correlations between e.s.d.'s in cell parameters are only used when they are defined by crystal symmetry. An approximate (isotropic) treatment of cell e.s.d.'s is used for estimating e.s.d.'s involving l.s. planes.
Refinement. Refinement of *F*^2^ against ALL reflections. The weighted *R*-factor *wR* and goodness of fit *S* are based on *F*^2^, conventional *R*-factors *R* are based on *F*, with *F* set to zero for negative *F*^2^. The threshold expression of *F*^2^ > σ(*F*^2^) is used only for calculating *R*-factors(gt) *etc*. and is not relevant to the choice of reflections for refinement. *R*-factors based on *F*^2^ are statistically about twice as large as those based on *F*, and *R*- factors based on ALL data will be even larger.

### Fractional atomic coordinates and isotropic or equivalent isotropic displacement parameters (Å^2^)

**Table:**

	*x*	*y*	*z*	*U*_iso_*/*U*_eq_	Occ. (<1)
Hg1	0.2500	0.529525 (12)	0.8750	0.01652 (3)	
Ni1	0.0000	0.5000	1.050596 (14)	0.01130 (8)	
S1	0.34284 (7)	0.39974 (6)	0.94163 (2)	0.01777 (12)	
S2	0.07460 (8)	0.66758 (7)	0.91464 (2)	0.02210 (14)	
C1	0.0628 (3)	0.7838 (2)	0.87530 (12)	0.0204 (4)	
C2	0.2196 (2)	0.4289 (2)	0.97739 (8)	0.0141 (4)	
N1	0.1356 (2)	0.4484 (2)	1.00263 (8)	0.0177 (4)	
N2	0.0514 (3)	0.8668 (2)	0.84917 (8)	0.0256 (5)	
O1	−0.0812 (2)	0.3139 (2)	1.05077 (16)	0.0593 (11)	
C3	−0.0338 (4)	0.1958 (4)	1.0511 (3)	0.075 (2)	
H2A	−0.1061	0.1322	1.0511	0.112*	0.50
H2B	0.0198	0.1834	1.0783	0.112*	0.50
H2C	0.0207	0.1825	1.0241	0.112*	0.50
H2D	0.0624	0.1999	1.0513	0.112*	0.50
H2E	−0.0635	0.1487	1.0240	0.112*	0.50
H2F	−0.0644	0.1495	1.0782	0.112*	0.50
H1	−0.164 (2)	0.331 (5)	1.0596 (16)	0.061 (14)*	

### Atomic displacement parameters (Å^2^)

**Table:**

	*U*^11^	*U*^22^	*U*^33^	*U*^12^	*U*^13^	*U*^23^
Hg1	0.02513 (7)	0.01081 (5)	0.01362 (5)	0.000	0.00653 (5)	0.000
Ni1	0.00883 (17)	0.01668 (19)	0.00839 (15)	0.00111 (13)	0.000	0.000
S1	0.0170 (3)	0.0176 (3)	0.0187 (3)	0.0054 (2)	0.0082 (2)	0.0037 (2)
S2	0.0323 (4)	0.0168 (3)	0.0173 (3)	0.0118 (3)	0.0094 (3)	0.0071 (2)
C1	0.0318 (12)	0.0153 (10)	0.0141 (9)	0.0070 (9)	0.0014 (11)	−0.0010 (10)
C2	0.0144 (10)	0.0145 (9)	0.0133 (10)	0.0015 (8)	−0.0006 (8)	0.0005 (8)
N1	0.0153 (9)	0.0255 (11)	0.0121 (8)	0.0009 (8)	−0.0008 (7)	−0.0011 (8)
N2	0.0478 (16)	0.0150 (10)	0.0141 (9)	0.0107 (10)	−0.0019 (10)	−0.0006 (8)
O1	0.0126 (10)	0.0178 (11)	0.147 (4)	0.0008 (8)	0.0117 (16)	0.0146 (16)
C3	0.030 (2)	0.0187 (16)	0.176 (7)	0.0003 (15)	−0.011 (3)	−0.011 (3)

### Geometric parameters (Å, º)

**Table:**

Hg1—S1^i^	2.5499 (7)	C1—N2	1.149 (4)
Hg1—S1	2.5499 (7)	C2—N1	1.151 (3)
Hg1—S2	2.5546 (7)	N2—Ni1^v^	2.041 (2)
Hg1—S2^i^	2.5546 (7)	O1—C3	1.295 (4)
Ni1—N2^ii^	2.041 (2)	O1—H1	0.897 (10)
Ni1—N2^iii^	2.041 (2)	C3—H2A	0.9800
Ni1—N1^iv^	2.045 (2)	C3—H2B	0.9800
Ni1—N1	2.045 (2)	C3—H2C	0.9800
Ni1—O1	2.066 (2)	C3—H2D	0.9800
Ni1—O1^iv^	2.066 (2)	C3—H2E	0.9800
S1—C2	1.666 (2)	C3—H2F	0.9800
S2—C1	1.661 (3)		
			
S1^i^—Hg1—S1	117.62 (3)	C1—N2—Ni1^v^	170.1 (3)
S1^i^—Hg1—S2	112.27 (2)	C3—O1—Ni1	134.5 (2)
S1—Hg1—S2	100.98 (2)	C3—O1—H1	122 (3)
S1^i^—Hg1—S2^i^	100.98 (2)	Ni1—O1—H1	101 (3)
S1—Hg1—S2^i^	112.27 (2)	O1—C3—H2A	109.5
S2—Hg1—S2^i^	113.29 (3)	O1—C3—H2B	109.5
N2^ii^—Ni1—N2^iii^	90.77 (13)	H2A—C3—H2B	109.5
N2^ii^—Ni1—N1^iv^	88.42 (9)	O1—C3—H2C	109.5
N2^iii^—Ni1—N1^iv^	179.17 (9)	H2A—C3—H2C	109.5
N2^ii^—Ni1—N1	179.17 (9)	H2B—C3—H2C	109.5
N2^iii^—Ni1—N1	88.42 (9)	O1—C3—H2D	109.5
N1^iv^—Ni1—N1	92.40 (12)	H2A—C3—H2D	141.1
N2^ii^—Ni1—O1	88.12 (13)	H2B—C3—H2D	56.3
N2^iii^—Ni1—O1	91.68 (14)	H2C—C3—H2D	56.3
N1^iv^—Ni1—O1	88.11 (13)	O1—C3—H2E	109.5
N1—Ni1—O1	92.08 (13)	H2A—C3—H2E	56.3
N2^ii^—Ni1—O1^iv^	91.68 (14)	H2B—C3—H2E	141.1
N2^iii^—Ni1—O1^iv^	88.12 (13)	H2C—C3—H2E	56.3
N1^iv^—Ni1—O1^iv^	92.08 (13)	H2D—C3—H2E	109.5
N1—Ni1—O1^iv^	88.11 (13)	O1—C3—H2F	109.5
O1—Ni1—O1^iv^	179.7 (3)	H2A—C3—H2F	56.3
C2—S1—Hg1	96.75 (9)	H2B—C3—H2F	56.3
C1—S2—Hg1	97.01 (10)	H2C—C3—H2F	141.1
N2—C1—S2	177.4 (3)	H2D—C3—H2F	109.5
N1—C2—S1	179.0 (2)	H2E—C3—H2F	109.5
C2—N1—Ni1	173.3 (2)		

Symmetry codes: (i) −*x*+1/2, *y*, −*z*+7/4; (ii) *y*−1, *x*+1/2, *z*+1/4; (iii) −*y*+1, −*x*+1/2, *z*+1/4; (iv) −*x*, −*y*+1, *z*; (v) −*y*+1/2, −*x*+1, *z*−1/4.

### Hydrogen-bond geometry (Å, º)

**Table:**

*D*—H···*A*	*D*—H	H···*A*	*D*···*A*	*D*—H···*A*
O1—H1···S1^vi^	0.90 (1)	2.40 (2)	3.262 (2)	160 (4)

Symmetry code: (vi) −*y*, *x*, −*z*+2.
